# Prevalence and Sources of Second-Hand Smoking Exposure among Non-Smoking Pregnant Women in an Urban Setting of Vietnam

**DOI:** 10.3390/ijerph16245022

**Published:** 2019-12-10

**Authors:** Chau Quy Ngo, Phuong Thu Phan, Giap Van Vu, Hanh Thi Chu, Toan Thi Nguyen, Mai Hong Nguyen, Hai Thanh Phan, Benjamin M. Y. Ong, Giang Thu Vu, Kiet Tuan Huy Pham, Bach Xuan Tran, Carl A. Latkin, Cyrus S. H. Ho, Roger C. M. Ho

**Affiliations:** 1 Department of Internal Medicine, Hanoi Medical University, Hanoi 100000, Vietnam; ngoquychaubmh@gmail.com (C.Q.N.); thuphuongdr@gmail.com (P.T.P.); vuvangiap@hmu.edu.vn (G.V.V.); nguyenthitoanmika.hmu@gmail.com (T.T.N.); nguyenhongmai1996@gmail.com (M.H.N.); 2 Respiratory Center, Bach Mai Hospital, Hanoi 100000, Vietnam; chuthihanhbmh@gmail.com; 3 Institute for Global Health Innovations, Duy Tan University, Da Nang 550000, Vietnam; 4 Yong Loo Lin School of Medicine, National University of Singapore, Singapore 119228, Singapore; e0012525@u.nus.edu; 5 Center of Excellence in Evidence-Based Medicine, Nguyen Tat Thanh University, Ho Chi Minh City 700000, Vietnam; giang.coentt@gmail.com; 6 Institute for Preventive Medicine and Public Health, Hanoi Medical University, Hanoi 100000, Vietnam; phamhuytuankiet_vkt@fpt.vn (K.T.H.P.); bach.ipmph@gmail.com (B.X.T.); 7 Bloomberg School of Public Health, Johns Hopkins University, Baltimore, MD 21205, USA; carl.latkin@jhu.edu; 8 Department of Psychological Medicine, National University Hospital, Singapore 119074, Singapore; cyrushosh@gmail.com; 9 Center of Excellence in Behavioral Medicine, Nguyen Tat Thanh University, Ho Chi Minh City 700000, Vietnam; pcmrhcm@nus.edu.sg; 10 Department of Psychological Medicine, Yong Loo Lin School of Medicine, National University of Singapore, Singapore 119228, Singapore; 11 Institute for Health Innovation and Technology (iHealthtech), National University of Singapore, Singapore 119077, Singapore

**Keywords:** second-hand smoking, pregnant women, urban, Vietnam

## Abstract

Exposure to second-hand smoke (SHS) among non-smoking pregnant women can lead to adverse maternal and fetal outcomes. A cross-sectional study was performed from July to August 2016 among 432 pregnant women at Bach Mai Hospital, Hanoi, Vietnam, to assess the prevalence and sources of SHS exposure among non-smoking pregnant women. Socio-economic characteristics and information regarding SHS exposure of participants were collected. Multivariable logistic regression was employed to identify associated factors. Overall, 92.6% and 64.5% of pregnant women were exposed to SHS in their lifetime and in the last 30 days, respectively. Cafeterias and restaurants had the highest proportion of pregnant women exposed to SHS. Those who reported that “smoking is allowed at home” (OR = 3.18; 95%CI = 1.97–5.13); going to working place (OR = 1.86; 95%CI = 1.08–3.19), going to state authority offices (OR = 1.98; 95%CI = 1.15–3.41), and cafeterias (OR = 1.96; 95%CI = 1.22–3.16) had the highest risk of SHS exposure in the last 30 days. We have found a high proportion of SHS exposure among non-smoking pregnant women in Vietnam. This comes from a multitude of sources, including homes, workplaces, cafeterias, and restaurants. The data emphasises the need for further intervention to address this health issue.

## 1. Introduction

Addressing SHS exposure in pregnant women is a critical public health issue if we are to improve maternal and fetal smoking well-being. Smoking and SHS exposure, also known as environmental tobacco smoke exposure or passive smoking, during pregnancy, is a significant risk factor for adverse pregnancy outcomes. It has been established that active smoking has adverse maternal and fetal outcomes, including, low birth weight [1,2], stillbirth and neonatal death [3], preterm premature rupture of membranes [4], placenta abruption [5,6], and placenta previa [7]. Recent studies have also found that SHS exposure contributes to adverse maternal and fetal outcome with increased risk of miscarriage [8], stillbirths [9], congenital malformations [9,10,11], lower mean birthweight [10], lung cancer, heart disease [12], and maternal depression [13].

Studies done throughout the world have shown vast differences in SHS exposure among pregnant women. The prevalence can be as high as 71.8% and 69.9% in Greece [14] and Southern India [15], respectively, or lower at 26.0% and 20.9% in Northern India [16] and Iran [17], respectively. Some factors that may contribute to decreased SHS exposure are higher maternal and paternal educational status [14], the presence of household smoking bans and the awareness of the harm of SHS exposure [18,19].

Interventions have been shown to be effective in reducing SHS exposure in pregnant women. For the issue of SHS exposure at home, interventions, such as simple advice and education given to women to help reduce their husband’s smoking frequency, was successful in reducing the number of cigarettes their husbands smoked and increased the attempts for them to stop smoking [20]. Transtheoretical model-based programmes aim to improve knowledge and self-efficacy among pregnant women and shows potential in reducing their SHS exposure [21]. One study among pregnant African American women showed that cognitive-behavioural intervention resulted in less SHS exposure before delivery and a significant improvement in very low birth weight and very preterm birth [22]. Thus, there is evidence to show that interventions can reduce SHS exposure in pregnant women and reduce adverse pregnancy outcomes. This reinforces the need to study the prevalence and sources of second-hand smoking in non-smoking women in Vietnam so as to ensure effective policies and interventions can be put in place.

Due to the apparent disease burden of SHS exposure in pregnant women, an international work group has made calls for more quantitative and qualitative data on exposure to SHS exposure in low to middle-income countries, which includes Vietnam [23]. The Global Adult Tobacco Survey [24] reported that the prevalence of SHS exposure among non-smokers was high in Vietnam with exposure at home at 67.6% and the workplace at 49.0%. With such a high prevalence of SHS exposure among non-smokers in Vietnam, we suspect that this could be an indicator that there is a high prevalence of SHS exposure among non-smoking pregnant women, and this would result in a larger disease burden and require active intervention. Data on smoking and SHS exposure has been recorded in Vietnam [24,25]. In particular, analysis of the 2008–2010 Global Adult Tobacco Survey (GATS) [26] showed that there is a high prevalence of SHS exposure at home among reproductive-aged women in Vietnam at 72.3%, which was the highest among 14 countries studied. They also noted that this was significantly higher among women living in rural areas compared with those living in urban areas. While existing information can provide some insight into the situation of SHS exposure among reproductive-aged women, there is a dearth in data specifically on pregnant women in this area. Furthermore, it is important to understand the different sources of SHS exposure to be able to implement effective interventional strategies. Thus, the aim of this study is to assess the prevalence and sources of SHS exposure among non-smoking pregnant women in Vietnam.

## 2. Materials and Methods 

### 2.1. Study Designs

We performed a cross-sectional study from July to August 2016 with 432 pregnant women at the Obstetrics Department of Bach Mai Hospital, Hanoi, Vietnam. The Bach Mai hospital is the largest general hospital in Vietnam. Annually, there are more than 6700 pregnant women visiting the Obstetrics Department for antenatal care. A convenient sampling method was used to recruit pregnant women to the study. They were invited to participate if they (1) were aged from 18 years old or above; (2) had sufficient capacities to answer the interview; and (3) agreed to give their written informed consents. The exclusion criterion was if the women were active smokers. Women from all periods of pregnancy were eligible as long as they met the other criteria. The approval of the Institutional Review Board was obtained through the Vietnam Respiratory Society.

### 2.2. Measurements

Women were approached by the data collectors who were medical students and nurses at the Bach Mai hospital. They were initially asked to identify the eligible criteria. After that, if they fulfilled the inclusion criteria, they were invited to a private room for the interview to assure their confidentiality and comfortability. They were introduced about the study purposes and their rights that they could withdraw from the study at any time without any influences on their current treatment and care. Women were then interviewed to collect information, including sociodemographic characteristics (age, education, occupation, and living location), gestation week, and whether they heard about second-hand smoking. 

Pregnant women were also asked to report: (1) Whether they were exposed to SHS during their lifetime (lifetime prevalence) and in the last 30 days (30-days prevalence); (2) Whether they allowed smoking at home and their exposure to SHS at home; (3) Whether they visited and were exposed to SHS at the workplace, state authority offices, health facilities, restaurants, cafeterias, public transports, schools, and non-smoking places (in general) in the last 30 days. 

### 2.3. Statistical Analysis

Stata software version 14.0 was used to analyse the data. Chi-squared test was utilised to compare the lifetime and 30-days prevalence among different sociodemographic characteristics. Multivariable logistic regression was employed to identify associated factors with second-hand smoking exposure in the last 30 days (Yes/No) as presented in odds ratio and confidence intervals. The potential associated factors included sociodemographic characteristics, ever hearing about second-hand smoking and whether they visited the workplace, state authority offices, health facilities, restaurants, cafeterias, public transports, and schools in the last 30 days. A p-value less than 0.05 was used to detect statistical significance.

### 2.4. Ethical Approval 

Study protocol was reviewed and approved by the Vietnam Respiratory Society Scientific and Ethics Committee (08-QD/VNRS).

## 3. Results

Among 432 pregnant women, Table 1 shows that 46.3% of them were aged from 26 to 30 years old. Most of the women had above high school levels of education (79.6%) and were employed (60.7%). The majority of respondents were living in urban areas (86.1%). There were 37.7% and 46.3% pregnant women at <30 gestation weeks and 30–37 gestation weeks, respectively. The majority of women had heard about second-hand smoking (65.1%), and 41.4% reported that smoking was allowed at their home.

**Figure 1:**
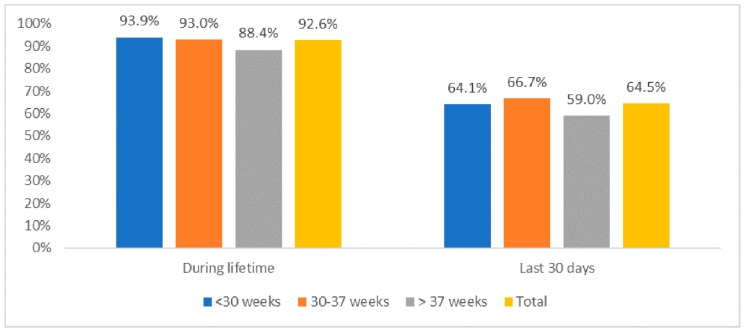
Life-time and 30-days prevalence of second-hand smoking exposure among pregnant women.

Figure 1 reveals that overall, 92.6% and 64.5% of pregnant women were exposed to SHS in their lifetime and in the last 30 days, respectively. There was no significant difference in the prevalence of second-hand smoking exposure among groups with different gestation weeks (*p* > 0.05).

**Figure 2:**
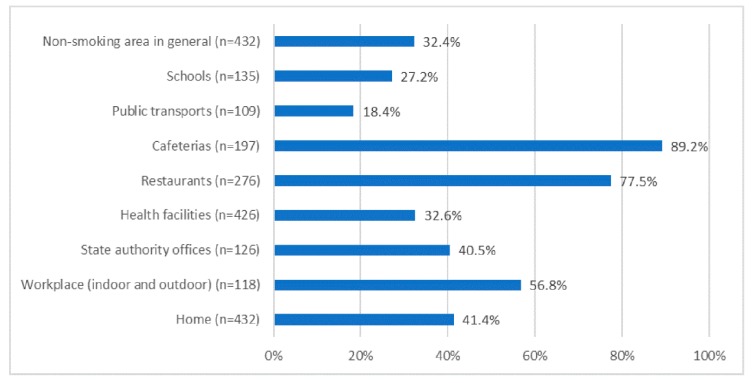
Environments where pregnant women were exposed to second-hand smoke in the last 30 days.

In the last 30 days, cafeterias and restaurants were the places where the highest proportion of pregnant women were exposed to SHS (89.2% and 77.5%, respectively), followed by the workplace (56.8%), home (41.4%), and state authority offices (40.5%) (Figure 2). 

Table 2 depicts the lifetime and 30-days prevalence of SHS exposure among pregnant women according to different sociodemographic characteristics. Statistical significance was only found in lifetime SHS exposure regarding educational attainment where higher educational attainment was associated with a higher prevalence of lifetime exposure to SHS. (*p* = 0.02).

Results of the adjusted regression model are presented in Table 3. Women with the highest risk consisted of those reporting that “smoking is allowed at home” (OR = 3.18; 95%CI = 1.97–5.13); going to the work place (OR = 1.86; 95%CI = 1.08–3.19), going to state authority offices (OR = 1.98; 95%CI = 1.15–3.41), and cafeterias (OR = 1.96; 95%CI = 1.22–3.16) in the last 30 days.

## 4. Discussion

In this study, we have found that 92.6% and 64.5% of pregnant women were exposed to SHS in their lifetime and in the last 30 days, respectively. This high proportion suggests that SHS exposure is an important health issue in Vietnam. Significant factors that contribute to SHS exposure within the last 30 days in non-smoking pregnant women are smoking being allowed at home, going to the workplace, state authority offices, and cafeterias or restaurants.

We found that smoking being allowed at home was associated with a significant increase in SHS exposure in non-smoking pregnant women. This is consistent with the Global Adult Tobacco Survey which reports that among non-smokers, the prevalence of SHS exposure in homes in Vietnam is 67.6%, which was the highest out of the 14 countries studied [24]. Addressing SHS exposure from family members who smoke is an important step to reduce the adverse outcomes of SHS exposure in pregnant women. A study done in rural China reports that paternal smoking was associated with a higher risk of spontaneous abortion (OR 1.17, 95% CI 1.16 to 1.19) [8]. Another study in Shanghai, China, reported that paternal smoking was associated with preterm birth (OR 1.18, 95% CI 0.98 to 1.43) [27]. However, the studies fail to determine causality—the results could possibly be due to the resultant passive smoking of the pregnant wife or the adverse effect of the smoking on sperm quality. Nonetheless, interventions to reduce SHS exposure at home have shown promise. In Guangzhou, China, simple advice and education were given to women to help reduce their husband’s smoking frequency, and this was successful in reducing the number of cigarettes their husbands smoked and increased the attempts for them to stop smoking [20]. It may be helpful to target both men and women with interventions as this will allow gender-sensitive tobacco control interventions and relieve the pressure off the woman to confront the male authority figure at home [28,29].

We also found that going to cafeterias and restaurants was associated with a significant increase in SHS exposure in non-smoking pregnant women. Cafeterias and restaurants were also the places with the highest proportion of pregnant women exposed to SHS (89.2% and 77.5%, respectively). Thus, it is important to explore measures to reduce SHS exposure at cafeterias and restaurants. A study in Canada reported that implementing no-smoking areas in bars and restaurants successfully reduced the probability of SHS exposure in up to 25% [30]. In North Dakota, United States of America, an 83% reduction in tobacco smoke pollution levels occurred after the passing of a comprehensive state-wide law prohibiting smoking in enclosed public places [31]. More legislation on restricting smoking at indoor venues, such as restaurants and cafeterias, appears to be an effective intervention in reducing SHS exposure.

We found that going to the workplace was associated with a significant increase in SHS exposure in non-smoking pregnant women. This is consistent with existing data that shows that there is a high prevalence of SHS exposure among women of reproductive age at the workplace at 40.7% [26]. In Finland, a national smoke-free legislation was shown to result in a clear decrease in employee SHS exposure and a significant decrease in median nicotine concentration at workplaces after one year [32]. Furthermore, smoke-free work policies have been shown to help smoking employees reduce the number of cigarettes smoked and eventually stop smoking [33]. This would lead to less SHS created by them at work and other places they may smoke at. Marcus and his colleagues (1992) demonstrated that more restrictive workplace smoking policies were associated with a lower proportion of non-smoking volunteers with detectable salivary cotinine [34]. SHS exposure at the workplace is an important issue to deal with as a large proportion of women are employed (60.7%). Future studies can consider assessing differences of SHS exposure between types of jobs and workplaces to further tailor interventions and policies.

The level of educational attainment was a statistically significant factor in relation to lifetime SHS exposure in non-smoking pregnant women where higher educational attainment was associated with a higher prevalence of lifetime exposure to SHS. This is contradictory to related literature where it was found that low education itself is related to a higher prevalence of smoking [35,36]. However, factors that could account for this difference is the difference in cultural setting, sampling from the hospital patients alone, and the small sample size of women with lower than high school level of education (*n* = 12). Further study looking specifically into the relationship between educational attainment and active and passive smoking in Vietnam would provide more insight into this area.

The Global Adult Tobacco Survey (GATS) [24] showed a significantly higher prevalence of second-hand smoke exposure among women in rural areas as compared to urban areas. However, our study found that there was no statistically significant difference in lifetime SHS exposure between these two groups. Possible reasons for this difference is that the GATS was conducted in a community setting where our study was conducted in a hospital setting. Furthermore, our study sampled pregnant women, where elsewhere, the GATS sampled women in general. The difference in study design could account for the non-significant difference in lifetime SHS exposure between pregnant women in the rural and urban areas. However, these findings indicate that pregnant women in rural and urban areas are equally at risk and interventions should be implemented to address both these areas. 

The high prevalence of SHS exposure among non-smoking pregnant women presents a heavy disease burden, and we suggest that more can be done to address this issue in the clinical and policy making setting. We suggest that clinicians can focus on proven methods, such as communicating with patients about the risks of and ways to avoid SHS exposure through simple advice and education [20]. The use of behaviour change interventions [37], provision of educational materials about SHS exposure, and counselling by an obstetrician [38] were found to be effective in decreasing SHS exposure in pregnant women. As for policy making, we suggest that implementing smoke-free legislation targeted at cafeterias and restaurants may help to alleviate the SHS exposure in non-smoking pregnant women. Such legislation, being total bans or the designation of smoke-free areas have been shown to reduce the prevalence of second-hand smoking [30,39]. Policies enforcing smoke-free workplaces have also shown to be effective in decreasing SHS exposure in workers [34,40,41]. To our knowledge, there are no existing effective smoking legislations implemented in Vietnam in recent times. Future interventional studies could consider working with law enforcement agencies to properly implement the policies and legislations.

The study was carried out by interviewing the patients after obtaining consent. The advantage of this was that it was a low-cost method, and the data was obtained rapidly and easily. However, the results relied on the participants’ self-reporting, which may result in recall bias due to the participants not remembering every instance they were exposed to SHS. Self-reporting also may lead to social desirability bias as the women may want to give answers that seem more socially acceptable and to avoid negative portrayal of themselves or their partners. For example, they may not want to admit that their husbands smoke at home as they do not want to put their husbands in a bad light. Moreover, they may report that they are non-smokers even though they do smoke out of fear of negative judgement. Thus, there may have been an under-reporting of smoking and SHS exposure. There may have been a sampling bias as the population was taken from a major hospital where the majority of the patients could come from more educated or urban backgrounds, which would not give an accurate representation of the less educated and those from rural backgrounds. Overall, these biases may result in an underestimation of SHS exposure in these non-smoking pregnant women and in turn, strengthen the interpretation that second-hand smoking in non-smoking pregnant women is a major health issue in Vietnam. This study was also limited by the fact that there was no qualitative data taken. This limits our insight into the social and cultural factors that may affect the SHS exposure. Our recommendation for future studies is to delve deeper into the specific social and cultural factors in Vietnam that may have a role to play in pregnant women’s exposure to SHS.

## 5. Conclusions

In conclusion, SHS exposure in non-smoking pregnant women is a major health issue in Vietnam with a large proportion of non-smoking women exposed to SHS exposure in the last 30 days (64.5%). We have found a high prevalence of SHS exposure from a multitude of sources, including homes, workplaces, and cafeterias and restaurants. The risk of adverse pregnancy outcomes due to SHS exposure during pregnancy is well documented. Thus, further studies are needed to evaluate the social and cultural aspects of this issue, and more interventions are needed to address this health issue. 

## Figures and Tables

**Table 1:** Demographic characteristics of pregnant women.

Characteristics	*n*	%
Age group		
18–25	118	27.3
26–30	200	46.3
31–35	79	18.3
>35	35	8.1
Educational attainment		
<High school	16	3.7
High school	72	16.7
>High school	344	79.6
Occupation		
Self-employed	120	27.8
Employed	262	60.7
Unemployed/Housewife	50	11.6
Living area		
Urban	372	86.1
Rural	60	13.9
Gestation week		
<30 weeks	163	37.7
30–37 weeks	200	46.3
>37 weeks	69	16.0
Ever heard about second-hand smoking	281	65.1
Smoking is allowed at home	179	41.4

**Table 2:** Lifetime and 30-days prevalence of second-hand smoking exposure among pregnant women according to different sociodemographic characteristics.

Characteristics	Lifetime Second-Hand Smoking Exposure			30-Days Second-Hand Smoking Exposure		
	*n*	%	*p*-Value	*n*	%	*p*-Value
Age group						
18–25	112	94.9	0.41	76	67.9	0.07
26–30	186	93.0		111	59.7	
31–35	70	88.6		53	75.7	
>35	32	91.4		18	56.3	
Educational attainment						
<High school	12	75.0	0.02	6	50.0	0.41
High school	68	94.4		47	69.1	
>High school	320	93.0		205	64.1	
Occupation						
Self-employed	110	91.7	0.87	71	64.6	0.74
Employed	244	93.1		155	63.5	
Unemployed/Housewife	46	92.0		32	69.6	
Living area						
Urban	345	92.7	0.77	221	64.1	0.64
Rural	55	91.7		37	67.3	
Gestation week						
<30 weeks	153	93.9	0.33	98	64.1	0.55
30–37 weeks	186	93.0		124	66.7	
>37 weeks	61	88.4		36	59.0	

**Table 3:** Factors associated with second-hand smoke exposure among pregnant women in the last 30 days.

Characteristics	Odds Ratio (OR) ^1^	*p*-Value	95% Confident Interval (CI)	
Age group (vs. 18–25)				
26–30	0.69	0.18	0.40	1.18
31–35	1.82	0.11	0.88	3.76
>35	0.52	0.14	0.21	1.24
Smoking is allowed at home (Yes vs. No)	3.18	<0.01	1.97	5.13
Going to working place in the last 30 days (Yes vs. No)	1.86	0.03	1.08	3.19
Going to any State authority offices in the last 30 days (Yes vs. No)	1.98	0.01	1.15	3.41
Going to any cafeterias in the last 30 days (Yes vs. No)	1.96	0.01	1.22	3.16
Going to any health facilities in the last 30 days (Yes vs. No)	9.02	0.07	0.87	93.09
Ever heard about second-hand smoking (Yes vs. No)	1.41	0.17	0.86	2.31

^1^ Full model also included: education, job, living area, gestation week, going to any restaurants in the last 30 days, using any public transports in the last 30 days, going to any schools in the last 30 days. Only variables with *p*-value <0.2 are presented.
